# Retroversion of the Gravid Uterus

**Published:** 1908-01-04

**Authors:** 


### GYNÆCOLOGY.

### RETROVERSION OF THE GRAVID UTERUS.

The subject of retroversion of the gravid uterus is one of great importance, and one of sufficiently frequent occurrence to be of interest to all. The symptoms produced by this condition are not as a rule clearly marked until either abortion is threatened or retention of urine occurs. When abortion is threatened, or actually occurs, it is caused not so much by the uterine displacement as by an endometritis, which often accompanies backward displacements of the uterus. Moreover, abortion occurs in these cases as a rule within the first two months, or, at all events, considerably before the time at which the uterus becomes large enough to cause retention of urine. Further, when the uterus has become large enough to cause retention of urine abortion does not often occur. This teaching is that of Herman, who has stated that out of a considerable number of these cases treated in the London Hospital none aborted. It cannot, however be said that abortion never occurs after retention of urine, because cases are known which prove the contrary.

Confining attention to cases which have gone on to retention of urine, the history related by the patient is generally very clear; as a rule they state that they have had a period of amenorrhœa of from three to four months' duration, and then with an accompaniment of backache and bearing down pain the urine begins to dribble away. This history is really almost diagnostic, but the fact that the patient says she is "constantly passing urine" is apt to mislead, and to lead to the belief that she has frequency of micturition.

The fact really is the opposite: the patient has retention of urine with overflow. If such a case is treated without a complete examination very serious results may happen. On examination of the patient a rounded fluctuating swelling is found in the mid-line of the abdomen reaching to a variable height, even to the ensiform cartilage. This is the distended bladder, and if any doubt of its identity exists it may be dispelled by the passage of the catheter.

On vaginal examination the cervix uteri will be found high up and displaced so that it looks towards the symphysis pubis, whilst in Douglas' pouch will be felt a soft, boggy, rounded swelling. In the face of the above-mentioned definite history with these findings there is seldom any doubt as to the diagnosis. It sometimes happens however, that the patient has had one or more slight irregular hæmorrhages, and that other signs of pregnancy are not definite. In such a case there may be a doubt as to the identity of the swelling in Douglas' pouch. On emptying the bladder, however, there should be no difficulty in establishing the fact that the second tumour is really the uterus. This becomes quite easy if the uterus can be pushed up out of Douglas' pouch and anteverted. Its character, consistence and shape will then as a rule make it clear that a pregnant uterus is being dealt with. If this condition remains undiagnosed, sooner or later the bladder becomes infected, even though no catheter has been passed. This happens the more readily because the resistance to infection is lowered by chronic over-distension. The results of infection of an over-distended bladder may be exceedingly severe; the whole mucous membrane of the bladder has been known to slough. The consequences to the uterus in untreated cases are of less importance, but incarceration may occur with the occasional production of abortion.

### Treatment.

Naturally the first thing to do is to empty the bladder. Scrupulous cleanliness and an aseptic catheter will insure this being done without risk of infection. It must not be forgotten that an over-distended is more easily infected than a normal bladder.

It is often necessary to pass the catheter in further than usual before the urine flows, because in these cases the neck of the bladder is rather stretched over the cervix and vagina than actually compressed by them. Indeed, the amount of compression is very slight, and it seems probable that retention of urine is caused in these cases more by some interference with the nervous mechanism of micturition than by mechanical obstacles. Enormous quantities of urine may be retained, even as much as five pints.

Having emptied the bladder the question arises, What is to be done with the uterus? The ideal procedure is to replace it bimanually at once, pushing up the fundus from the posterior fornix, or from the rectum, and making use of the genu-pectoral position as an aid in this manœuvre. If this can be done without causing great pain the uterus can be easily kept in place by a ring pessary, and no further trouble ensues. This replacement cannot always be effected at once, and then several courses are open to us. According to Herman, if the patient is kept in bed, and the bladder empty, the uterus always rights itself.

In hospital this method is very successful, and may be carried out in private with equal success if the catheter can be passed at least every eight hours. According to Sinclair, the uterus will right itself after once emptying the bladder if a large ring pessary is placed in the vagina and the patient kept in bed lying on her side.

Occasionally it is necessary to give an anæsthetic to replace the uterus if the above-mentioned more simple methods should fail: but they very rarely do fail. In an occasional case it occurs that the uterus is bound down by inflammatory adhesions, and becoming pregnant causes retention of urine. In such a case it is impossible to replace the uterus, nor will it right itself if the bladder be kept empty. The only possible treatment is to open the abdomen, separate adhesions, and lift the uterus up out of the pelvis. The diagnosis of this condition is very difficult, but the severity of the symptoms would probably suggest laparotomy on other grounds.

